# Ethyl 1-benzyl-3-(4-bromo­phen­yl)-1*H*-pyrazole-5-carboxyl­ate

**DOI:** 10.1107/S1600536811024986

**Published:** 2011-07-06

**Authors:** Jiong Jia, He Yang, Yan Qing Ge, Jian Wu Wang

**Affiliations:** a School of Chemistry and Chemical Engineering, Shandong University, Jinan 250100, People’s Republic of China

## Abstract

In the title compound, C_19_H_17_BrN_2_O_2_, the pyrazole ring makes dihedral angles of 88.00 (16) and 5.78 (13)° with the phenyl and bromo­phenyl rings, respectively. In the crystal, mol­ecules are linked by weak inter­molecular C—H⋯O hydrogen bonds.

## Related literature

For the pharmacological activity of pyrazole compounds and applications of nitro­gen-containing heterocyclic compounds, see: Ge *et al.* (2009 ▶, 2011 ▶). For the related structures, see: Han *et al.* (2011 ▶); Ge *et al.* (2007 ▶); Li *et al.* (2011 ▶).
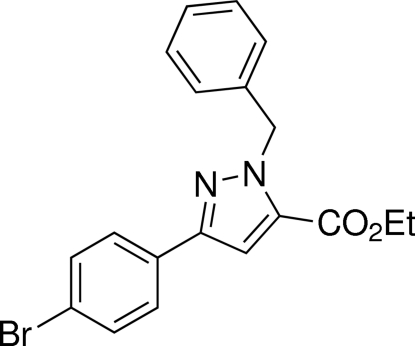


## Experimental

### 

#### Crystal data

C_19_H_17_BrN_2_O_2_
*M* *_r_* = 385.26
Monoclinic, 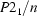

*a* = 10.5656 (13) Å
*b* = 15.3433 (19) Å
*c* = 11.5706 (14) Å
β = 111.506 (2)°
*V* = 1745.1 (4) Å^3^
*Z* = 4
Mo *K*α radiation
μ = 2.37 mm^−1^
*T* = 298 K
0.22 × 0.16 × 0.12 mm

#### Data collection

Bruker SMART CCD area-detector diffractometer
Absorption correction: multi-scan (*SADABS*; Sheldrick, 1996) ▶ *T*_min_ = 0.624, *T*_max_ = 0.764
8955 measured reflections
3089 independent reflections
2332 reflections with *I* > 2σ(*I*)
*R*_int_ = 0.022

#### Refinement

*R*[*F* ^2^ > 2σ(*F* ^2^)] = 0.034
*wR*(*F* ^2^) = 0.088
*S* = 1.02
3089 reflections
217 parameters
H-atom parameters constrained
Δρ_max_ = 0.33 e Å^−3^
Δρ_min_ = −0.70 e Å^−3^

### 

Data collection: *SMART* (Bruker, 2005 ▶); cell refinement: *SAINT* (Bruker, 2005 ▶); data reduction: *SAINT*; program(s) used to solve structure: *SHELXS97* (Sheldrick, 2008 ▶); program(s) used to refine structure: *SHELXL97* (Sheldrick, 2008 ▶); molecular graphics: *XP* in *SHELXTL* (Sheldrick, 2008 ▶); software used to prepare material for publication: *SHELXL97*.

## Supplementary Material

Crystal structure: contains datablock(s) I, global. DOI: 10.1107/S1600536811024986/wn2438sup1.cif

Structure factors: contains datablock(s) I. DOI: 10.1107/S1600536811024986/wn2438Isup2.hkl

Supplementary material file. DOI: 10.1107/S1600536811024986/wn2438Isup3.cml

Additional supplementary materials:  crystallographic information; 3D view; checkCIF report

## Figures and Tables

**Table 1:** Hydrogen-bond geometry (Å, °)

*D*—H⋯*A*	*D*—H	H⋯*A*	*D*⋯*A*	*D*—H⋯*A*
C16—H16⋯O1^i^	0.93	2.50	3.369 (4)	155

Symmetry code: (i) 
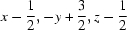
.

**Fig. 1.**
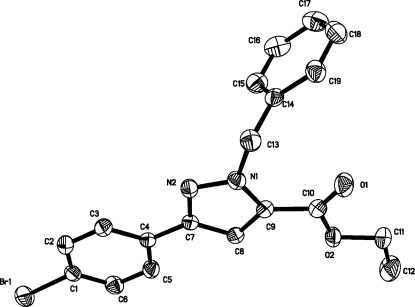
The molecular structure of the title compound, showing displacement ellipsoids drawn at the 50% probability level. H atoms have been omitted.

**Fig. 2.**
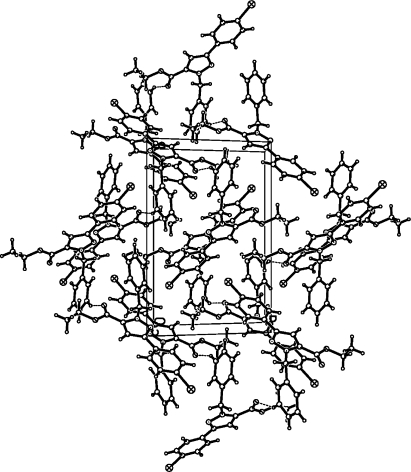
A view of the packing of molecules in the crystal structure. Dashed lines indicate hydrogen bonds.

## supplementary crystallographic information

### Comment

Synthesis of nitrogen-containing heterocyclic compounds has been a subject of great interest due to their wide application in agrochemical and pharmaceutical fields (Ge *et al.,*  2009, 2011). Some pyrazole derivatives which belong to this category have been of interest for their biological activities. Considerable efforts have been devoted to the development of novel pyrazole compounds.

We report here the crystal structure of the title compound (Fig. 1). The pyrazole ring makes dihedral angles of 88.00 (16)° and 5.78 (13)° with the phenyl and bromophenyl rings, respectively. In the fluorophenyl analogue (Han *et al.,* 2011) the corresponding angles are 81.19 (18) and 4.57 (16)°. In the tolyl analogue (Li *et al.,* 2011) the corresponding angles are 83.40 (4) and 15.68 (4)°. The crystal structure of another related compound has been reported (Ge *et al.,* 2007). In the crystal structure, molecules are linked by weak intermolecular C—H···O hydrogen bonds.

### Experimental

A mixture of ethyl 3-(4-bromophenyl)-1*H*-pyrazole-5-carboxylate (0.02 mol), (chloromethyl)benzene (0.0024 mol) and potassium carbonate (0.02 mol) in acetonitrile (100 ml) was heated to reflux for 5 h. The solvent was removed under reduced pressure and a product was isolated by column chromatography on silica gel (yield 81%). Crystals of the title compound suitable for X-ray diffraction were obtained by allowing a refluxed solution of the product in ethyl acetate to cool slowly to room temperature and allowing the solvent to evaporate over a period of 2 d.

### Refinement

All hydrogen atoms were positioned geometrically [C—H = 0.93 Å for Csp^2^, C—H = 0.97 Å for methylene C and C—H = 0.96 Å for methyl C] and were refined using a riding model, with *U*_iso_(H) = x*U*_eq_(C), where x = 1.5 for methyl H and 1.2 for all other H atoms.

### Crystal data

**Table:**

C_19_H_17_BrN_2_O_2_	*F*(000) = 784
*M**_r_* = 385.26	*D*_x_ = 1.466 Mg m^−^^3^
Monoclinic, *P*2_1_/*n*	Mo *K*α radiation, λ = 0.71073 Å
Hall symbol: -P 2yn	Cell parameters from 3103 reflections
*a* = 10.5656 (13) Å	θ = 2.2–25.3°
*b* = 15.3433 (19) Å	µ = 2.37 mm^−^^1^
*c* = 11.5706 (14) Å	*T* = 298 K
β = 111.506 (2)°	Block, colorless
*V* = 1745.1 (4) Å^3^	0.22 × 0.16 × 0.12 mm
*Z* = 4	

### Data collection

**Table:**

Bruker SMART CCD area-detector diffractometer	3089 independent reflections
Radiation source: fine-focus sealed tube	2332 reflections with *I* > 2σ(*I*)
graphite	*R*_int_ = 0.022
phi and ω scans	θ_max_ = 25.1°, θ_min_ = 2.2°
Absorption correction: multi-scan (*SADABS*; Sheldrick, 1996)	*h* = −12→12
*T*_min_ = 0.624, *T*_max_ = 0.764	*k* = −18→17
8955 measured reflections	*l* = −13→11

### Refinement

**Table:**

Refinement on *F*^2^	Primary atom site location: structure-invariant direct methods
Least-squares matrix: full	Secondary atom site location: difference Fourier map
*R*[*F*^2^ > 2σ(*F*^2^)] = 0.034	Hydrogen site location: inferred from neighbouring sites
*wR*(*F*^2^) = 0.088	H-atom parameters constrained
*S* = 1.02	*w* = 1/[σ^2^(*F*_o_^2^) + (0.0415*P*)^2^ + 0.8018*P*] where *P* = (*F*_o_^2^ + 2*F*_c_^2^)/3
3089 reflections	(Δ/σ)_max_ < 0.001
217 parameters	Δρ_max_ = 0.33 e Å^−^^3^
0 restraints	Δρ_min_ = −0.70 e Å^−^^3^

### Special details

**Table:**

Geometry. All e.s.d.'s (except the e.s.d. in the dihedral angle between two l.s. planes) are estimated using the full covariance matrix. The cell e.s.d.'s are taken into account individually in the estimation of e.s.d.'s in distances, angles and torsion angles; correlations between e.s.d.'s in cell parameters are only used when they are defined by crystal symmetry. An approximate (isotropic) treatment of cell e.s.d.'s is used for estimating e.s.d.'s involving l.s. planes.
Refinement. Refinement of *F*^2^ against ALL reflections. The weighted *R*-factor *wR* and goodness of fit *S* are based on *F*^2^, conventional *R*-factors *R* are based on *F*, with *F* set to zero for negative *F*^2^. The threshold expression of *F*^2^ > σ(*F*^2^) is used only for calculating *R*-factors(gt) *etc*. and is not relevant to the choice of reflections for refinement. *R*-factors based on *F*^2^ are statistically about twice as large as those based on *F*, and *R*- factors based on ALL data will be even larger.

### Fractional atomic coordinates and isotropic or equivalent isotropic displacement parameters (Å^2^)

**Table:**

	*x*	*y*	*z*	*U*_iso_*/*U*_eq_	
Br1	0.14954 (3)	0.23557 (2)	0.33861 (3)	0.07268 (15)	
N1	0.5993 (2)	0.57103 (14)	0.88561 (19)	0.0464 (5)	
N2	0.4883 (2)	0.52724 (13)	0.81156 (19)	0.0458 (5)	
O1	0.87411 (19)	0.63973 (15)	1.00922 (19)	0.0689 (6)	
O2	0.92884 (17)	0.57135 (12)	0.86274 (18)	0.0552 (5)	
C1	0.2646 (3)	0.31434 (16)	0.4583 (2)	0.0460 (6)	
C2	0.2191 (3)	0.35288 (18)	0.5429 (3)	0.0540 (7)	
H2	0.1316	0.3421	0.5405	0.065*	
C3	0.3052 (3)	0.40806 (17)	0.6317 (3)	0.0495 (6)	
H3	0.2748	0.4342	0.6892	0.059*	
C4	0.4364 (2)	0.42517 (14)	0.6366 (2)	0.0385 (5)	
C5	0.4785 (3)	0.38498 (17)	0.5496 (2)	0.0501 (6)	
H5	0.5657	0.3955	0.5509	0.060*	
C6	0.3933 (3)	0.32961 (18)	0.4609 (3)	0.0540 (7)	
H6	0.4231	0.3029	0.4033	0.065*	
C7	0.5294 (2)	0.48270 (15)	0.7316 (2)	0.0391 (5)	
C8	0.6665 (2)	0.49904 (16)	0.7551 (2)	0.0425 (6)	
H8	0.7185	0.4762	0.7126	0.051*	
C9	0.7089 (2)	0.55554 (16)	0.8535 (2)	0.0428 (6)	
C10	0.8439 (3)	0.59442 (17)	0.9187 (3)	0.0482 (6)	
C11	1.0643 (3)	0.6089 (2)	0.9142 (3)	0.0624 (8)	
H11A	1.1091	0.5912	1.0002	0.075*	
H11B	1.0590	0.6721	0.9111	0.075*	
C12	1.1416 (3)	0.5767 (3)	0.8380 (4)	0.0874 (12)	
H12A	1.1463	0.5142	0.8421	0.131*	
H12B	1.2319	0.6004	0.8696	0.131*	
H12C	1.0963	0.5946	0.7533	0.131*	
C13	0.5871 (3)	0.62843 (18)	0.9823 (3)	0.0534 (7)	
H13A	0.6578	0.6137	1.0610	0.064*	
H13B	0.5000	0.6180	0.9900	0.064*	
C14	0.5983 (2)	0.72389 (17)	0.9565 (2)	0.0447 (6)	
C15	0.5372 (3)	0.7587 (2)	0.8389 (3)	0.0596 (8)	
H15	0.4890	0.7225	0.7730	0.071*	
C16	0.5469 (4)	0.8471 (2)	0.8179 (3)	0.0712 (9)	
H16	0.5064	0.8695	0.7381	0.085*	
C17	0.6163 (4)	0.9013 (2)	0.9148 (3)	0.0719 (9)	
H17	0.6217	0.9607	0.9010	0.086*	
C18	0.6778 (3)	0.8676 (2)	1.0323 (3)	0.0677 (9)	
H18	0.7254	0.9041	1.0981	0.081*	
C19	0.6687 (3)	0.77974 (19)	1.0524 (3)	0.0551 (7)	
H19	0.7108	0.7575	1.1321	0.066*	

### Atomic displacement parameters (Å^2^)

**Table:**

	*U*^11^	*U*^22^	*U*^33^	*U*^12^	*U*^13^	*U*^23^
Br1	0.0671 (2)	0.0617 (2)	0.0738 (2)	−0.01026 (15)	0.00757 (17)	−0.02063 (16)
N1	0.0462 (12)	0.0457 (12)	0.0486 (13)	−0.0028 (10)	0.0190 (10)	−0.0077 (10)
N2	0.0414 (12)	0.0458 (12)	0.0506 (13)	−0.0048 (9)	0.0172 (10)	−0.0060 (10)
O1	0.0518 (12)	0.0864 (16)	0.0595 (13)	−0.0039 (11)	0.0099 (10)	−0.0261 (12)
O2	0.0386 (10)	0.0590 (12)	0.0668 (12)	−0.0089 (8)	0.0182 (9)	−0.0150 (9)
C1	0.0467 (15)	0.0361 (14)	0.0478 (15)	−0.0021 (11)	0.0084 (12)	−0.0007 (11)
C2	0.0434 (15)	0.0511 (16)	0.0680 (18)	−0.0061 (12)	0.0210 (14)	−0.0079 (13)
C3	0.0442 (15)	0.0488 (15)	0.0588 (17)	0.0000 (12)	0.0228 (13)	−0.0062 (13)
C4	0.0376 (13)	0.0327 (12)	0.0434 (14)	0.0027 (10)	0.0128 (11)	0.0044 (10)
C5	0.0447 (15)	0.0530 (16)	0.0555 (16)	−0.0042 (12)	0.0221 (13)	−0.0065 (13)
C6	0.0620 (18)	0.0511 (16)	0.0534 (17)	−0.0033 (13)	0.0263 (14)	−0.0100 (13)
C7	0.0390 (13)	0.0329 (12)	0.0447 (14)	0.0017 (10)	0.0146 (11)	0.0020 (10)
C8	0.0398 (14)	0.0411 (14)	0.0489 (15)	0.0010 (11)	0.0190 (12)	−0.0037 (11)
C9	0.0384 (13)	0.0422 (14)	0.0472 (15)	0.0021 (11)	0.0152 (11)	0.0003 (11)
C10	0.0438 (15)	0.0464 (15)	0.0495 (16)	0.0022 (12)	0.0111 (13)	−0.0001 (13)
C11	0.0400 (15)	0.0645 (19)	0.075 (2)	−0.0077 (13)	0.0121 (14)	−0.0046 (15)
C12	0.055 (2)	0.107 (3)	0.110 (3)	−0.0105 (19)	0.041 (2)	−0.007 (2)
C13	0.0558 (17)	0.0602 (17)	0.0497 (16)	−0.0044 (14)	0.0259 (14)	−0.0095 (13)
C14	0.0397 (14)	0.0543 (16)	0.0442 (15)	0.0028 (11)	0.0202 (12)	−0.0090 (12)
C15	0.0621 (19)	0.070 (2)	0.0467 (17)	0.0058 (15)	0.0196 (14)	−0.0094 (14)
C16	0.086 (2)	0.076 (2)	0.0583 (19)	0.0211 (19)	0.0350 (18)	0.0132 (17)
C17	0.093 (2)	0.0549 (19)	0.091 (3)	0.0002 (17)	0.061 (2)	0.0007 (18)
C18	0.072 (2)	0.061 (2)	0.078 (2)	−0.0158 (16)	0.0364 (18)	−0.0192 (17)
C19	0.0540 (17)	0.0639 (19)	0.0467 (16)	−0.0058 (14)	0.0176 (13)	−0.0096 (13)

### Geometric parameters (Å, °)

**Table:**

Br1—C1	1.903 (2)	C9—C10	1.474 (4)
N1—N2	1.351 (3)	C11—C12	1.489 (4)
N1—C9	1.360 (3)	C11—H11A	0.9700
N1—C13	1.466 (3)	C11—H11B	0.9700
N2—C7	1.344 (3)	C12—H12A	0.9600
O1—C10	1.199 (3)	C12—H12B	0.9600
O2—C10	1.332 (3)	C12—H12C	0.9600
O2—C11	1.453 (3)	C13—C14	1.508 (4)
C1—C6	1.369 (4)	C13—H13A	0.9700
C1—C2	1.371 (4)	C13—H13B	0.9700
C2—C3	1.384 (4)	C14—C15	1.382 (4)
C2—H2	0.9300	C14—C19	1.383 (4)
C3—C4	1.391 (3)	C15—C16	1.387 (4)
C3—H3	0.9300	C15—H15	0.9300
C4—C5	1.386 (3)	C16—C17	1.373 (5)
C4—C7	1.470 (3)	C16—H16	0.9300
C5—C6	1.381 (4)	C17—C18	1.375 (5)
C5—H5	0.9300	C17—H17	0.9300
C6—H6	0.9300	C18—C19	1.378 (4)
C7—C8	1.395 (3)	C18—H18	0.9300
C8—C9	1.369 (3)	C19—H19	0.9300
C8—H8	0.9300		
			
N2—N1—C9	111.58 (19)	O2—C11—H11A	110.3
N2—N1—C13	118.9 (2)	C12—C11—H11A	110.3
C9—N1—C13	129.5 (2)	O2—C11—H11B	110.3
C7—N2—N1	105.40 (19)	C12—C11—H11B	110.3
C10—O2—C11	115.6 (2)	H11A—C11—H11B	108.5
C6—C1—C2	121.0 (2)	C11—C12—H12A	109.5
C6—C1—Br1	119.2 (2)	C11—C12—H12B	109.5
C2—C1—Br1	119.7 (2)	H12A—C12—H12B	109.5
C1—C2—C3	119.1 (2)	C11—C12—H12C	109.5
C1—C2—H2	120.4	H12A—C12—H12C	109.5
C3—C2—H2	120.4	H12B—C12—H12C	109.5
C2—C3—C4	121.3 (2)	N1—C13—C14	113.4 (2)
C2—C3—H3	119.3	N1—C13—H13A	108.9
C4—C3—H3	119.3	C14—C13—H13A	108.9
C5—C4—C3	117.8 (2)	N1—C13—H13B	108.9
C5—C4—C7	120.4 (2)	C14—C13—H13B	108.9
C3—C4—C7	121.8 (2)	H13A—C13—H13B	107.7
C6—C5—C4	121.2 (2)	C15—C14—C19	118.0 (3)
C6—C5—H5	119.4	C15—C14—C13	121.9 (2)
C4—C5—H5	119.4	C19—C14—C13	120.1 (3)
C1—C6—C5	119.5 (3)	C14—C15—C16	120.9 (3)
C1—C6—H6	120.2	C14—C15—H15	119.6
C5—C6—H6	120.2	C16—C15—H15	119.6
N2—C7—C8	110.4 (2)	C17—C16—C15	120.0 (3)
N2—C7—C4	121.6 (2)	C17—C16—H16	120.0
C8—C7—C4	128.0 (2)	C15—C16—H16	120.0
C9—C8—C7	106.0 (2)	C16—C17—C18	119.8 (3)
C9—C8—H8	127.0	C16—C17—H17	120.1
C7—C8—H8	127.0	C18—C17—H17	120.1
N1—C9—C8	106.6 (2)	C17—C18—C19	119.9 (3)
N1—C9—C10	123.4 (2)	C17—C18—H18	120.1
C8—C9—C10	130.0 (2)	C19—C18—H18	120.1
O1—C10—O2	124.6 (2)	C18—C19—C14	121.4 (3)
O1—C10—C9	125.4 (3)	C18—C19—H19	119.3
O2—C10—C9	110.0 (2)	C14—C19—H19	119.3
O2—C11—C12	107.3 (2)		
			
C9—N1—N2—C7	0.4 (3)	C13—N1—C9—C10	2.5 (4)
C13—N1—N2—C7	178.5 (2)	C7—C8—C9—N1	0.1 (3)
C6—C1—C2—C3	0.1 (4)	C7—C8—C9—C10	179.4 (3)
Br1—C1—C2—C3	178.2 (2)	C11—O2—C10—O1	−3.4 (4)
C1—C2—C3—C4	0.1 (4)	C11—O2—C10—C9	177.1 (2)
C2—C3—C4—C5	−0.1 (4)	N1—C9—C10—O1	3.8 (4)
C2—C3—C4—C7	−179.1 (2)	C8—C9—C10—O1	−175.4 (3)
C3—C4—C5—C6	−0.1 (4)	N1—C9—C10—O2	−176.6 (2)
C7—C4—C5—C6	178.9 (2)	C8—C9—C10—O2	4.1 (4)
C2—C1—C6—C5	−0.3 (4)	C10—O2—C11—C12	−179.0 (3)
Br1—C1—C6—C5	−178.4 (2)	N2—N1—C13—C14	−110.3 (3)
C4—C5—C6—C1	0.3 (4)	C9—N1—C13—C14	67.4 (4)
N1—N2—C7—C8	−0.4 (3)	N1—C13—C14—C15	40.9 (4)
N1—N2—C7—C4	178.8 (2)	N1—C13—C14—C19	−140.5 (2)
C5—C4—C7—N2	175.3 (2)	C19—C14—C15—C16	0.2 (4)
C3—C4—C7—N2	−5.7 (4)	C13—C14—C15—C16	178.9 (3)
C5—C4—C7—C8	−5.7 (4)	C14—C15—C16—C17	−0.9 (5)
C3—C4—C7—C8	173.3 (2)	C15—C16—C17—C18	0.9 (5)
N2—C7—C8—C9	0.2 (3)	C16—C17—C18—C19	−0.4 (5)
C4—C7—C8—C9	−178.9 (2)	C17—C18—C19—C14	−0.2 (5)
N2—N1—C9—C8	−0.3 (3)	C15—C14—C19—C18	0.3 (4)
C13—N1—C9—C8	−178.1 (2)	C13—C14—C19—C18	−178.4 (3)
N2—N1—C9—C10	−179.7 (2)		

### Hydrogen-bond geometry (Å, °)

**Table:**

*D*—H···*A*	*D*—H	H···*A*	*D*···*A*	*D*—H···*A*
C16—H16···O1^i^	0.93	2.50	3.369 (4)	155

Symmetry codes: (i) *x*−1/2, −*y*+3/2, *z*−1/2.
